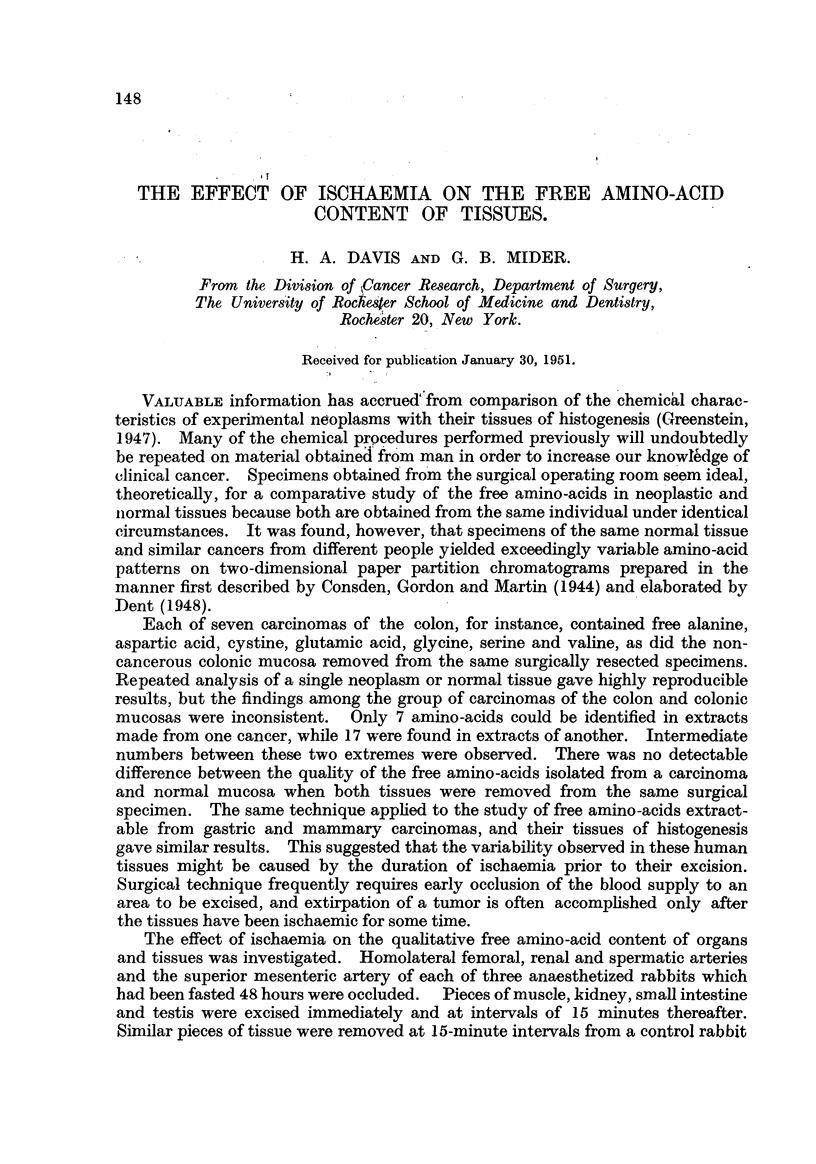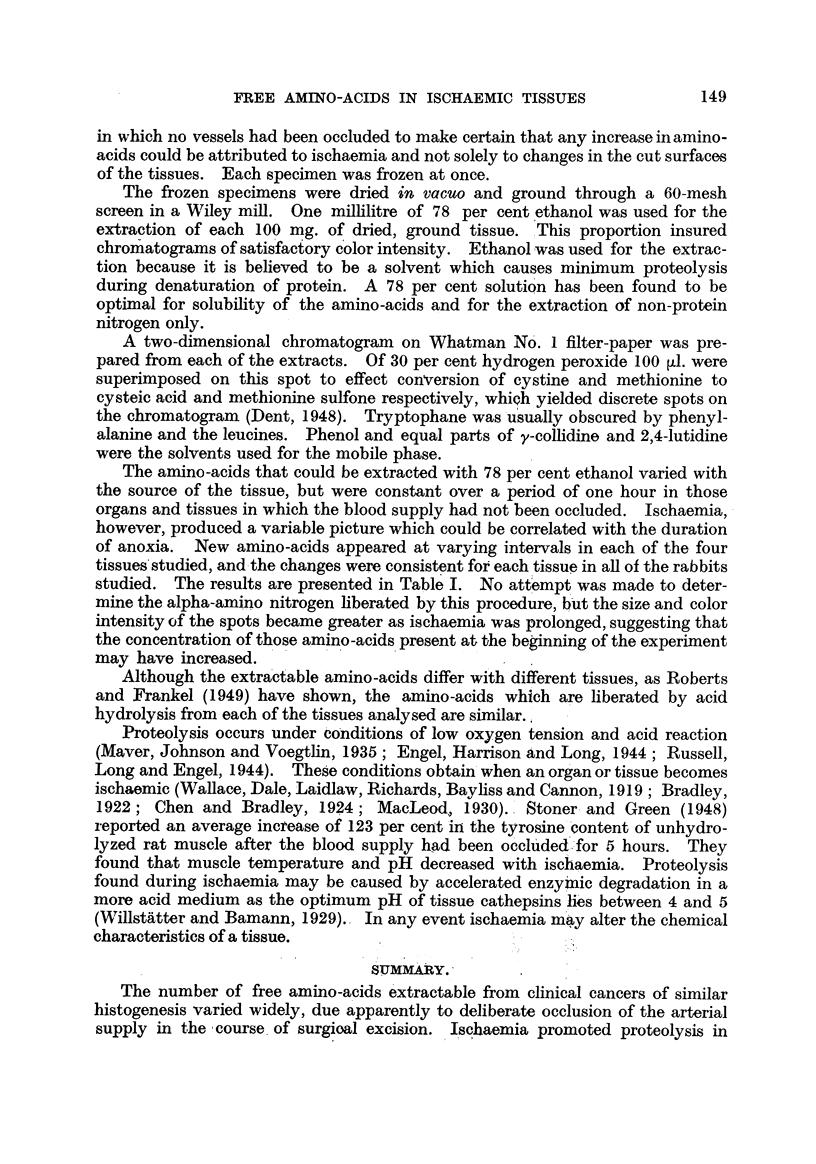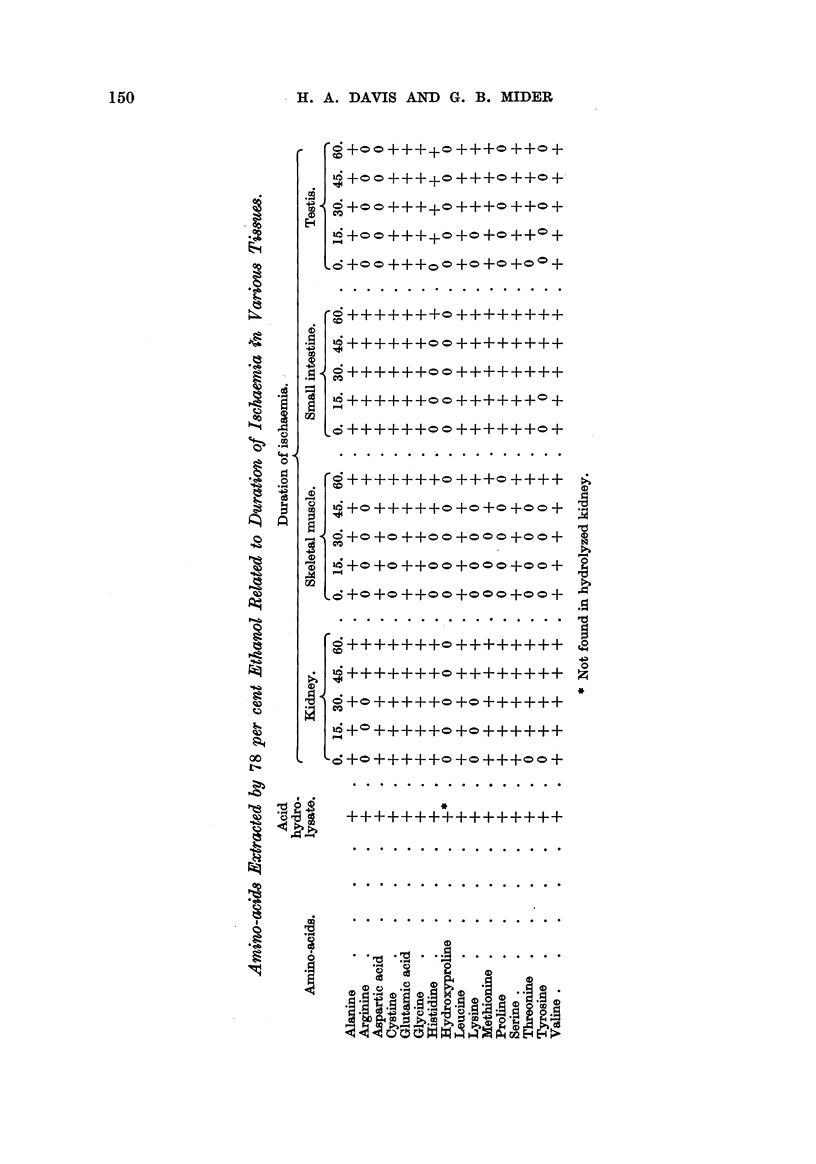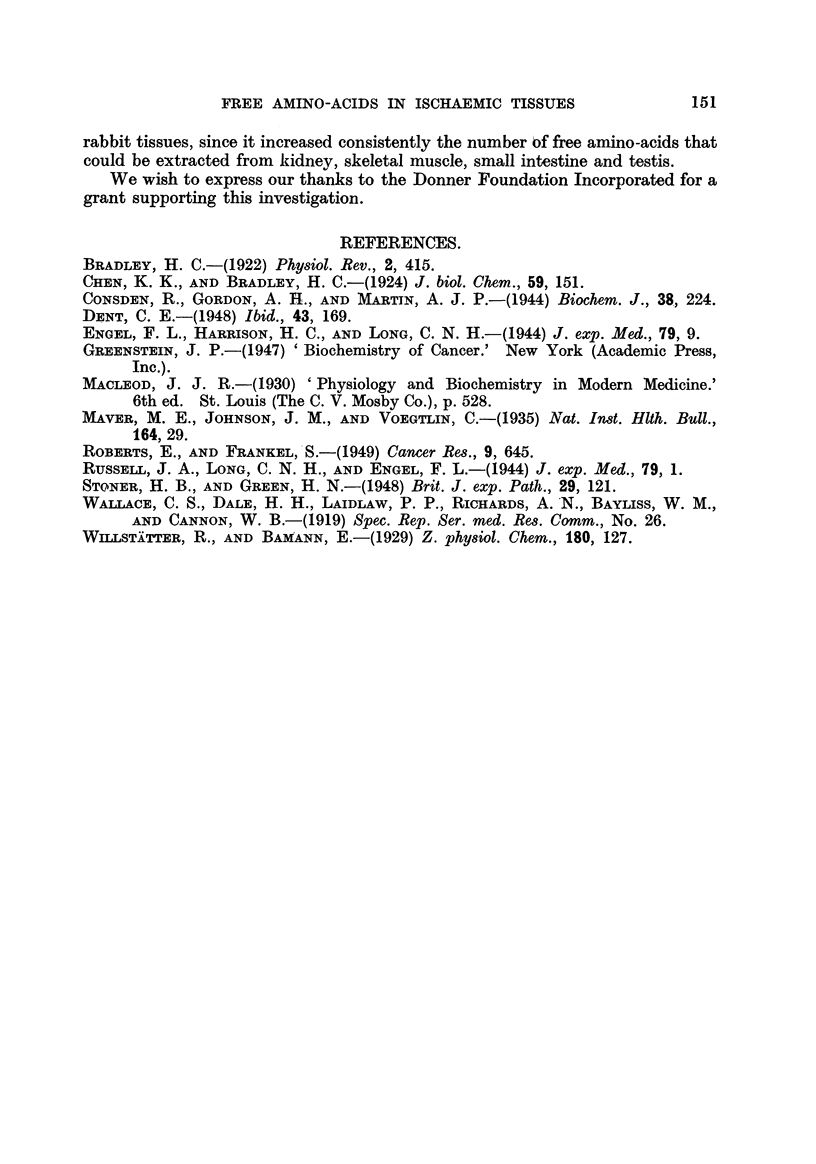# The Effect of Ischaemia on the Free Amino-acid Content of Tissues

**DOI:** 10.1038/bjc.1951.15

**Published:** 1951-03

**Authors:** H. A. Davis, G. B. Mider


### THE EFFECT OF ISCHAEMIA ON THE FREE AMINO-ACID CONTENT OF TISSUES.

H. A. DAVIS AND G. B. MIDER.

*From the Division of Cancer Research, Department of Surgery, The University of Rochester School of Medicine and Dentistry, Rochester 20, New York.*

Received for publication January 30, 1951.

VALUABLE information has accrued from comparison of the chemical characteristics of experimental neoplasms with their tissues of histogenesis (Greenstein, 1947). Many of the chemical procedures performed previously will undoubtedly be repeated on material obtained from man in order to increase our knowledge of clinical cancer. Specimens obtained from the surgical operating room seem ideal, theoretically, for a comparative study of the free amino-acids in neoplastic and normal tissues because both are obtained from the same individual under identical circumstances. It was found, however, that specimens of the same normal tissue and similar cancers from different people yielded exceedingly variable amino-acid patterns on two-dimensional paper partition chromatograms prepared in the manner first described by Consden, Gordon and Martin (1944) and elaborated by Dent (1948).

Each of seven carcinomas of the colon, for instance, contained free alanine, aspartic acid, cystine, glutamic acid, glycine, serine and valine, as did the non-cancerous colonic mucosa removed from the same surgically resected specimens. Repeated analysis of a single neoplasm or normal tissue gave highly reproducible results, but the findings among the group of carcinomas of the colon and colonic mucosas were inconsistent. Only 7 amino-acids could be identified in extracts made from one cancer, while 17 were found in extracts of another. Intermediate numbers between these two extremes were observed. There was no detectable difference between the quality of the free amino-acids isolated from a carcinoma and normal mucosa when both tissues were removed from the same surgical specimen. The same technique applied to the study of free amino-acids extractable from gastric and mammary carcinomas, and their tissues of histogenesis gave similar results. This suggested that the variability observed in these human tissues might be caused by the duration of ischaemia prior to their excision. Surgical technique frequently requires early occlusion of the blood supply to an area to be excised, and extirpation of a tumor is often accomplished only after the tissues have been ischaemic for some time.

The effect of ischaemia on the qualitative free amino-acid content of organs and tissues was investigated. Homolateral femoral, renal and spermatic arteries and the superior mesenteric artery of each of three anaesthetized rabbits which had been fasted 48 hours were occluded. Pieces of muscle, kidney, small intestine and testis were excised immediately and at intervals of 15 minutes thereafter. Similar pieces of tissue were removed at 15-minute intervals from a control rabbit in which no vessels had been occluded to make certain that any increase in amino-acids could be attributed to ischaemia and not solely to changes in the cut surfaces of the tissues. Each specimen was frozen at once.

The frozen specimens were dried *in vacuo* and ground through a 60-mesh screen in a Wiley mill. One millilitre of 78 per cent ethanol was used for the extraction of each 100 mg. of dried, ground tissue. This proportion insured chromatograms of satisfactory color intensity. Ethanol was used for the extraction because it is believed to be a solvent which causes minimum proteolysis during denaturation of protein. A 78 per cent solution has been found to be optimal for solubility of the amino-acids and for the extraction of non-protein nitrogen only.

A two-dimensional chromatogram on Whatman No. 1 filter-paper was prepared from each of the extracts. Of 30 per cent hydrogen peroxide 100 $\mu$l. were superimposed on this spot to effect conversion of cystine and methionine to cysteic acid and methionine sulfone respectively, which yielded discrete spots on the chromatogram (Dent, 1948). Tryptophane was usually obscured by phenylalanine and the leucines. Phenol and equal parts of $\gamma$-collidine and 2,4-lutidine were the solvents used for the mobile phase.

The amino-acids that could be extracted with 78 per cent ethanol varied with the source of the tissue, but were constant over a period of one hour in those organs and tissues in which the blood supply had not been occluded. Ischaemia, however, produced a variable picture which could be correlated with the duration of anoxia. New amino-acids appeared at varying intervals in each of the four tissues studied, and the changes were consistent for each tissue in all of the rabbits studied. The results are presented in Table I. No attempt was made to determine the alpha-amino nitrogen liberated by this procedure, but the size and color intensity of the spots became greater as ischaemia was prolonged, suggesting that the concentration of those amino-acids present at the beginning of the experiment may have increased.

Although the extractable amino-acids differ with different tissues, as Roberts and Frankel (1949) have shown, the amino-acids which are liberated by acid hydrolysis from each of the tissues analysed are similar.

Proteolysis occurs under conditions of low oxygen tension and acid reaction (Maver, Johnson and Voegtlin, 1935 ; Engel, Harrison and Long, 1944 ; Russell, Long and Engel, 1944). These conditions obtain when an organ or tissue becomes ischaemic (Wallace, Dale, Laidlaw, Richards, Bayliss and Cannon, 1919 ; Bradley, 1922 ; Chen and Bradley, 1924 ; MacLeod, 1930). Stoner and Green (1948) reported an average increase of 123 per cent in the tyrosine content of unhydrolyzed rat muscle after the blood supply had been occluded for 5 hours. They found that muscle temperature and pH decreased with ischaemia. Proteolysis found during ischaemia may be caused by accelerated enzymic degradation in a more acid medium as the optimum pH of tissue cathepsins lies between 4 and 5 (Willstätter and Bamann, 1929). In any event ischaemia may alter the chemical characteristics of a tissue.

### SUMMARY.

The number of free amino-acids extractable from clinical cancers of similar histogenesis varied widely, due apparently to deliberate occlusion of the arterial supply in the course of surgical excision. Ischaemia promoted proteolysis in

*Amino-acids Extracted by 78 per cent Ethanol Related to Duration of Ischaemia in Various Tissues.*

| Amino-acids. | Acid hydrolysate. | Duration of ischaemia. Kidney. 0. | 15. | 30. | 45. | 60. | Skeletal muscle. 0. | 15. | 30. | 45. | 60. | Small intestine. 0. | 15. | 30. | 45. | 60. | Testis. 0. | 15. | 30. | 45. | 60. |
|---|---|---|---|---|---|---|---|---|---|---|---|---|---|---|---|---|---|---|---|---|---|
| Alanine | + | + | + | + | + | + | + | + | + | + | + | + | + | + | + | + | + | + | + | + | + |
| Arginine | + | 0 | 0 | 0 | + | + | 0 | 0 | 0 | 0 | + | + | + | + | + | + | 0 | 0 | 0 | 0 | 0 |
| Aspartic acid | + | + | + | + | + | + | + | + | + | + | + | + | + | + | + | + | 0 | 0 | 0 | 0 | 0 |
| Cystine | + | + | + | + | + | + | 0 | 0 | 0 | + | + | + | + | + | + | + | + | + | + | + | + |
| Glutamic acid | + | + | + | + | + | + | + | + | + | + | + | + | + | + | + | + | + | + | + | + | + |
| Glycine | + | + | + | + | + | + | + | + | + | + | + | + | + | + | + | + | + | + | + | + | + |
| Histidine | + | + | + | + | + | + | 0 | 0 | 0 | + | + | 0 | 0 | 0 | 0 | + | 0 | + | + | + | + |
| Hydroxyproline | +* | 0 | 0 | 0 | 0 | 0 | 0 | 0 | 0 | 0 | 0 | 0 | 0 | 0 | 0 | 0 | 0 | 0 | 0 | 0 | 0 |
| Leucine | + | + | + | + | + | + | + | + | + | + | + | + | + | + | + | + | + | + | + | + | + |
| Lysine | + | 0 | 0 | 0 | + | + | 0 | 0 | 0 | 0 | + | + | + | + | + | + | 0 | 0 | + | + | + |
| Methionine | + | + | + | + | + | + | 0 | 0 | 0 | + | + | + | + | + | + | + | + | + | + | + | + |
| Proline | + | + | + | + | + | + | 0 | 0 | 0 | 0 | 0 | + | + | + | + | + | 0 | 0 | 0 | 0 | 0 |
| Serine | + | + | + | + | + | + | + | + | + | + | + | + | + | + | + | + | + | + | + | + | + |
| Threonine | + | 0 | + | + | + | + | 0 | 0 | 0 | 0 | + | + | + | + | + | + | 0 | + | + | + | + |
| Tyrosine | + | 0 | + | + | + | + | 0 | 0 | 0 | 0 | + | 0 | 0 | + | + | + | 0 | 0 | 0 | 0 | 0 |
| Valine | + | + | + | + | + | + | + | + | + | + | + | + | + | + | + | + | + | + | + | + | + |

* Not found in hydrolyzed kidney.

rabbit tissues, since it increased consistently the number of free amino-acids that could be extracted from kidney, skeletal muscle, small intestine and testis.

We wish to express our thanks to the Donner Foundation Incorporated for a grant supporting this investigation.

### REFERENCES.

Bradley, H. C.—(1922) *Physiol. Rev.*, **2**, 415.

Chen, K. K., and Bradley, H. C.—(1924) *J. biol. Chem.*, **59**, 151.

Consden, R., Gordon, A. H., and Martin, A. J. P.—(1944) *Biochem. J.*, **38**, 224.

Dent, C. E.—(1948) *Ibid.*, **43**, 169.

Engel, F. L., Harrison, H. C., and Long, C. N. H.—(1944) *J. exp. Med.*, **79**, 9.

Greenstein, J. P.—(1947) 'Biochemistry of Cancer.' New York (Academic Press, Inc.).

Macleod, J. J. R.—(1930) 'Physiology and Biochemistry in Modern Medicine.' 6th ed. St. Louis (The C. V. Mosby Co.), p. 528.

Maver, M. E., Johnson, J. M., and Voegtlin, C.—(1935) *Nat. Inst. Hlth. Bull.*, **164**, 29.

Roberts, E., and Frankel, S.—(1949) *Cancer Res.*, **9**, 645.

Russell, J. A., Long, C. N. H., and Engel, F. L.—(1944) *J. exp. Med.*, **79**, 1.

Stoner, H. B., and Green, H. N.—(1948) *Brit. J. exp. Path.*, **29**, 121.

Wallace, C. S., Dale, H. H., Laidlaw, P. P., Richards, A. N., Bayliss, W. M., and Cannon, W. B.—(1919) *Spec. Rep. Ser. med. Res. Comm.*, No. 26.

Willstätter, R., and Bamann, E.—(1929) *Z. physiol. Chem.*, **180**, 127.